# Highly sensitive flexible heat flux sensor based on a microhole array for ultralow to high temperatures

**DOI:** 10.1038/s41378-023-00599-9

**Published:** 2023-10-24

**Authors:** Le Li, Bian Tian, Zhongkai Zhang, Meng Shi, Jiangjiang Liu, Zhaojun Liu, Jiaming Lei, Shuimin Li, Qijing Lin, Libo Zhao, Zhuangde Jiang

**Affiliations:** 1 School of Mechanical Engineering, Xi’an Jiaotong University, 710049 Xi’an, China; 2 State Key Laboratory for Manufacturing Systems Engineering, International Joint Laboratory for Micro/Nano Manufacturing and Measurement Technologies, Xi’an Jiaotong University (Yantai) Research Institute for Intelligent Sensing Technology and System, Xi’an Jiaotong University, Xi’an, 710049 China; 3 Shandong Laboratory of Yantai Advanced Materials and Green Manufacturing, Yantai, 265503 China

**Keywords:** Electronic devices, Sensors

## Abstract

With the growing demand for thermal management of electronic devices, cooling of high-precision instruments, and biological cryopreservation, heat flux measurement of complex surfaces and at ultralow temperatures has become highly imperative. However, current heat flux sensors (HFSs) are commonly used in high-temperature scenarios and have problems when applied in low-temperature conditions, such as low sensitivity and embrittlement. In this study, we developed a flexible and highly sensitive HFS that can operate at ultralow to high temperatures, ranging from −196 °C to 273 °C. The sensitivities of HFSs with thicknesses of 0.2 mm and 0.3 mm, which are efficiently manufactured by the screen-printing method, reach 11.21 μV/(W/m^2^) and 13.43 μV/(W/m^2^), respectively. The experimental results show that there is a less than 3% resistance change from bending to stretching. Additionally, the HFS can measure heat flux in both exothermic and absorptive cases and can measure heat flux up to 25 kW/m^2^. Additionally, we demonstrate the application of the HFS to the measurement of minuscule heat flux, such as heat dissipation of human skin and cold water. This technology is expected to be used in heat flux measurements at ultralow temperatures or on complex surfaces, which has great importance in the superconductor and cryobiology field.

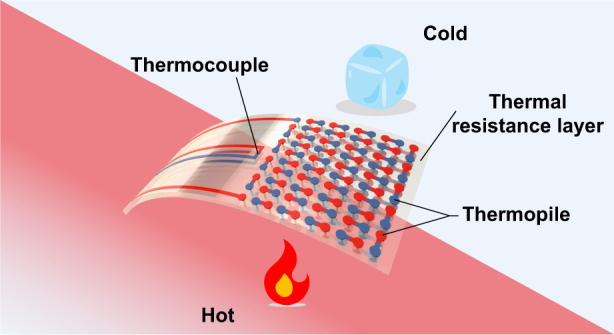

## Introduction

Heat flux measurement is essential in the fields of meteorology^1^, device heat dissipation^2–4^, and building insulation^5^. However, recent studies in thermal metrology commonly focus more on temperature change measurement^6,7^ and less on heat flux measurement when analyzing the heat transfer process. The temperature reflects the degree of cold and heat of the object, and the heat flux reflects the strength of heat exchange during the heat transfer process. There is a large amount of heat exchange between high- and low-temperature environments, and measuring the temperature alone cannot reflect the dynamic process during heating or cooling^8^. Therefore, it is necessary to measure the heat flux, especially in ultralow- and high-temperature scenarios. Existing HFSs, such as the Gardon gauge^9,10^, coaxial thermocouple^11,12^, and Schmidt-Boelter type heat flux meter^13,14^, are complex in structure, large in size, or have low sensitivity coefficients for heat flux. Water cooling devices or heat sinks usually need to be added for the Gardon gauge, resulting in a large volume. It can be used for the measurement of heat radiation and heat convection, while it has difficulty measuring curved surfaces^15,16^. The coaxial thermocouple has strong scour resistance, but the sensitivity is low, and the measurement error is large for small heat flux^17^. The Schmidt-Boelter heat flux meter uses a thermocouple wire around a thermal resistance layer to form a thermopile, and the time lag of the sensor is large^18^. Thin-film heat flux sensors have thermopiles in thin film form, which are currently popular for heat flux measurement due to their small size, easy installation, and large measurement range^19,20^. However, existing studies focus more on HFSs used at high temperatures^21^ and less on devices that are flexible, bendable, and easy to attach and install. Li et al.^22,23^ studied HFSs for aeroengines with a sensitivity of 0.06193 μV/(W/m^2^), which could measure 236.4 kW/m^2^ at 888 °C through an anti-emission coating, ultimately achieving a sensitivity of 0.04856 μV/(W/m^2^). Fu et al.^24^ developed an HFS able to survive 1 h in 1000 °C air with a sensitivity of 0.0038 μV/(W/m^2^). Wang et al.^25^ proposed a new thermopile-type HFS based on the printed circuit board (PCB) process, with a sensitivity of 0.2670 μV/(W/m^2^). Since these sensors were prepared on rigid substrates, they could not meet the flexibility requirements and had low sensitivity. Hayriye Gidik et al.^26^ prepared six distinct types of heat flux meters for fabrics. Although flexibility was achieved with a heat flux sensitivity of 2.46 μV/(W/m^2^), a thickness of 1.19 mm, and a thermal resistance of 0.039 m^2^ KW^−1^, the excessive thickness and thermal resistance affected the heat transfer process, which reduced the accuracy of the heat flux measurement.

A thermoresistive HFS needs to measure the temperature gradient. The thermocouple, due to its high-accuracy temperature measurement, can be made into a thin layer, reducing its impact on the heat transfer process; moreover, it has excellent linearity and a quick response time^27^. Thermocouples or thermopiles are prepared by the electron beam evaporation method^28,29^, magnetron sputtering method^30,31^, etc. The preparation process is complex, and it’s difficult to form specific patterns. For example, the magnetron sputtering and electron beam evaporation methods require complex equipment and a strict vacuum environment^32,33^. The film formation speed is slow; usually, only the micron level can be reached with several hours of preparation. The patterning of the film is usually completed by photolithography. By comparison, screen printing, using a slurry and a mask screen plate, can complete the preparation and patterning of sensitive films. The film thickness in a single printing can reach several microns to tens of microns, and the preparation is efficient and fast and has been used to produce a variety of devices^34–36^. It has also been used to produce thermoelectric devices with high Seebeck coefficients and a wide temperature range^37,38^. The required materials can be easily doped using screen printing^39^, making this method conducive to the study of the effects of the materials on the thermoelectric potential output properties.

To achieve more accurate heat flux measurements, it is necessary to raise the thermal potential output of the HFS while reducing the total thickness of the sensor^40^. In this work, by punching small holes in the substrate to connect thermopiles on the upper and lower surfaces in series, we designed a new HFS structure with a significant reduction in the total thickness. Indium tin oxide (ITO) and indium oxide (In_2_O_3_) materials were selected as thermoelectric materials. These materials have a greater thermoelectric output than conventional metallic thermocouples^41,42^, maintain chemical stability at high and low temperatures^43,44^ and have a Seebeck coefficient of up to 224 μV/°C^45^. The preparation of the thermopile was carried out using screen printing. We measured its microscopic morphology and built a thermal flux sensor calibration bench. The resulting test revealed excellent performance parameters and resistance stability at various bending radii. Additional tests also evaluated exothermic or heat absorption phenomena such as hot water heat dissipation, ultralow-temperature liquid nitrogen volatilization, human skin heat dissipation, and heat gun application.

## Materials and methods

### Sensor design

**Fig. 1:**
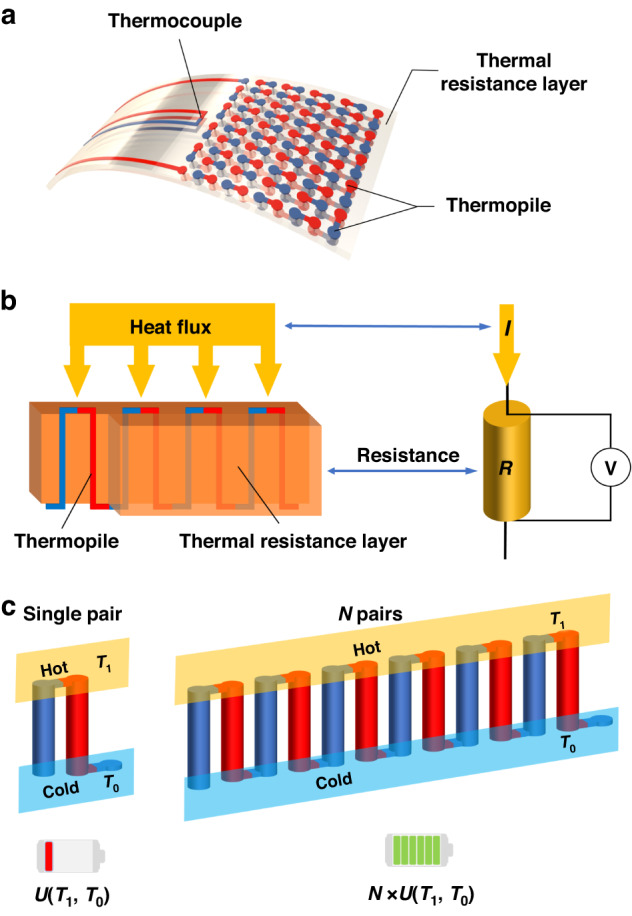
Design of the HFS. **a** Structure of the designed sensor with integrated HFS and temperature sensors, where the HFS includes a thermoelectric layer, a thermal resistance layer, and temperature sensors distributed on the upper and lower surfaces of the substrate. **b** Principle of the thermal resistance HFS, which is similar to Ohm’s law in electricity. **c** Difference between a thermopile and a single thermocouple. When the thermopile consists of *N* pairs of thermocouples connected in series, the output electric potential of the thermopile is *N* times the output electric potential of a single pair of thermocouples

Figure 1a shows the overall diagram of the sensor, which consists of a flexible polyimide (PI) substrate, a thermopile and two thermocouples. The thermopile is used to measure heat flux, and the thermocouples on the front and back sides are used to measure temperature. Polyimide acts as the sensor substrate and the thermal resistance layer in the heat transfer process. Fifty pairs of thermocouples connected in series comprise the thermopile. Figure 1b illustrates the principle of heat flux measurement, which is based on Fourier’s law. Eq. (1) shows the calculation of the heat flux.

1 $$q=\lambda \frac{{dt}}{{dx}}=\lambda \frac{\Delta T}{\Delta x}$$

where *q* (W/m^2^) is the heat, *λ* (W/(m·K)) is the thermal conductivity of the thermal resistance layer, and *dT*/*dx* is the temperature gradient in the direction of the thickness of the thermal resistance layer. When the thermal resistance layer is sufficiently thin, *dT*/*dx* is equivalent to Δ*T*/Δ*x*.

Similar to Ohm’s law, thermal resistance is analogous to resistance, temperature is analogous to voltage, and heat flux is analogous to current. The current in a circuit can be obtained by measuring the voltage between the ends of the resistance area divided by the resistance. In the heat flux measurement, the heat flux is equal to the difference in the temperature at the ends of the thermal resistance layer divided by the thermal resistance. The thermal resistance, similar to resistance, is related to the thermal conductivity and thickness of the material. The thermal resistance is usually a known quantity. To avoid the sensor being too large for heat transfer, the thermal resistance layer is generally made into a thin layer, which makes it more difficult to measure the temperature difference between the upper and lower surfaces. The use of multiple pairs of thermocouples in series to form a thermopile can amplify the temperature difference signal. Figure 1c shows the principle of a thermopile. The thermal electromotive force generated by a single pair of thermocouples is a function of the temperature difference between the hot end and the cold end, and by forming a thermopile with *N* pairs in series, the output electric potential will become *N* times that of a single pair of thermocouples. Half of the nodes in the thermopile are located in the high-temperature region, and the other half of the nodes are located in the low-temperature region. The electric potential is calculated as in Eq. (2),

2 $$E=N\,\cdot\, S\,\cdot\, \Delta T$$

where *E* (V) is the electric potential, *N* is the number of thermocouples connected in series, *S* (V/K) is the Seebeck coefficient of the thermocouple, and Δ*T* is the temperature difference between the hot and cold ends of the thermocouple. The combination of Eq. (1) and Eq. (2) produces the calculation of the heat flux, as shown in Eq. (3).

3 $$q=\frac{Q}{A}=\frac{\lambda }{\Delta x\,\cdot\, S\,\cdot\, N}E$$

Once the sensor is prepared, the thickness and thermal conductivity of the thermal resistance layer, the Seebeck coefficient of the thermocouple, and the number of thermocouples in series of the thermopile are determined. Only the thermoelectric potential of the sensor needs to be measured, then the heat flux can still be calculated. When there is heat transfer to the sensor, a high-temperature zone is formed on the upper surface of the HFS. The temperature of the bottom surface of the sensor is lower than that of the upper surface due to the substrate barrier, thus producing a temperature difference. The sensitivity coefficient of the HFS can be calculated by Eq. (4).

4 $$C=\frac{E}{q}=\frac{\Delta x\,\cdot\, S\,\cdot\, N}{\lambda }$$

where *C* (V/(W/m^2^)) is the sensitivity coefficient of the HFS. It can be concluded that the sensitivity of the sensor can be improved by increasing the thickness of the thermal resistance layer, the Seebeck coefficient, and the number of thermocouples in series and choosing materials with lower thermal conductivity as the thermal resistance layer.

The traditional thermal resistance layer of an HFS is usually a thin layer prepared by magnetron sputtering and other processes, usually less than 1 μm thick. Here, the use of the substrate as a thermal resistance layer greatly improves its thickness. The HFS thermal resistance interferes with the heat transfer process, making the heat flux measurement inaccurate. Compared to the previous structure, this structure does not incorporate a new thermal resistance, so the measurement accuracy of the sensor is guaranteed. Here, we choose PI as the substrate, and its thermal conductivity is 0.22 (W/(m·K)). Using ITO-In_2_O_3_ as a thermally sensitive material, the Seebeck coefficient of the oxide has a great advantage compared to the traditional metal thermocouple, further improving the sensitivity of the sensor.

**Fig. 2:**
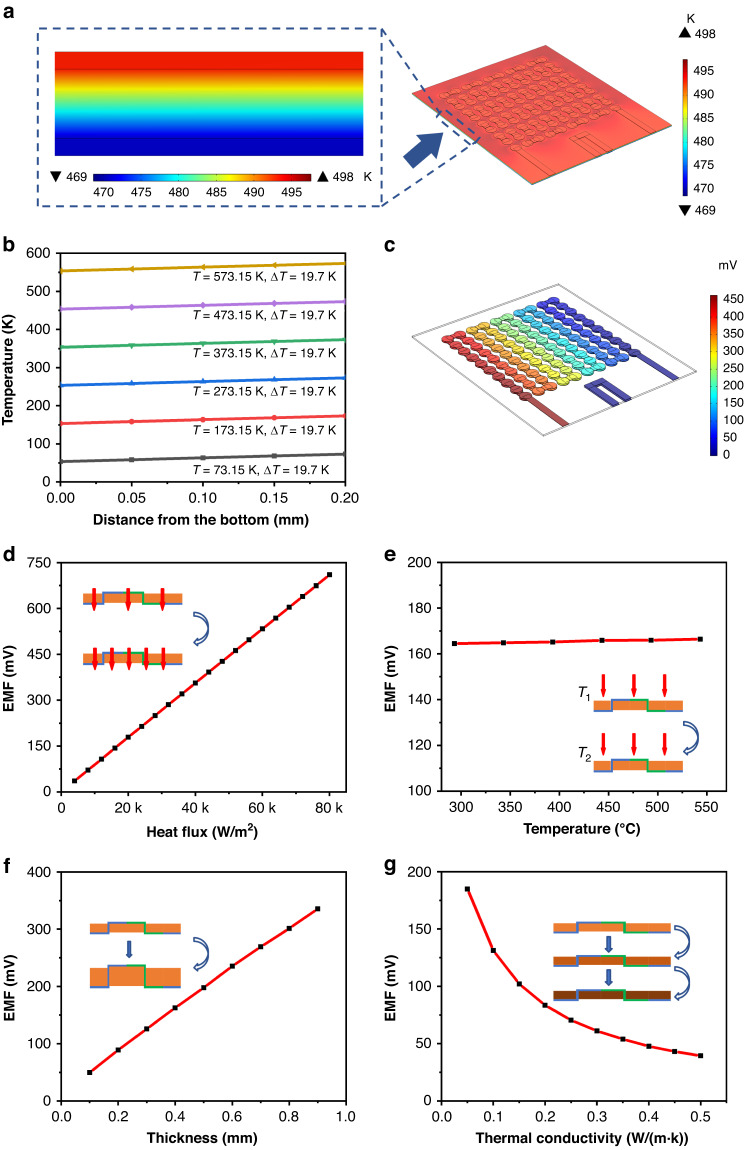
Simulative analysis of the HFS. **a** Cloud plot of the simulated temperature distribution of the HFS. The temperature shows an increasing trend from the bottom to the top. **b** Temperature distribution inside the thermal resistance layer at different temperatures. The temperature difference between the top and the bottom is always kept at 19.7 K, and the temperature gradient inside the thermal resistance layer remains constant. **c** Cloud plot of the thermal potential output. The thermal potential gradually changes from one end of the thermopile to the other. **d** The HFS sensor output voltage and heat flux show a linear increasing relationship. **e** The HFS output voltage does not vary with temperature. **f** The HFS output voltage increases with the thickness of the thermal resistance layer, showing a linear increasing relationship. **g** The HFS output voltage decreases with increasing thermal conductivity of the thermal resistance layer

Figure 2 shows the simulation calculation results of the designed sensor, Fig. S1 shows the sensor simulation calculation process, and Table S1 shows the main geometric parameters of the sensor. The COMSOL Multiphysics software package (Version 5.6, COMSOL Inc., Stockholm, Sweden) was used to build the sensor model, and Fig. 2a shows the temperature distribution cloud of the sensor. The temperature on the upper surface of the HFS remains constant. The temperature decreases along the thickness direction from the upper surface to the lower surface, from 498 K to 469 K. Since the thermal conductivity of the thermoelectric layer is much larger than that of the thermal resistance layer and the thickness of the thermoelectric layer is smaller than that of the thermal resistance layer, the temperature change is mainly concentrated in the thermal resistance layer, thus avoiding any negative influence of the thermopile on heat transfer. Figure 2b shows the temperature at the nodes every 0.05 mm from the lower surface to the upper surface of the sensor. When the heat flux through the sensor is fixed and the temperature is raised from 73.15 K to 573.15 K, the temperature gradient between the upper and lower surfaces does not change even with changes in the thickness of the HFS. The temperature difference is always 19.7 K, independent of the surface temperature magnitude. Figure 2c shows the thermal potential output cloud of the sensor. The potential of the thermopile increases from the initial end to the terminal; the set condition is a temperature of 493.15 K, and the heat flux is 52 kW/m^2^. The thermal potential output is more than 450 mV showing a high sensitivity to the heat flux. Figure 2d–g shows the relationship between the output electromotive force and the heat flux, temperature, thermal resistance layer thickness and thermal conductivity. The sensor output increases as the heat flux increases. The sensor output voltage shows a linear correlation with the heat flux; when the temperature changes, the output of the HFS changes only minutely, consistent with the conclusion that the temperature difference along the thickness direction is constant. Moreover, when the temperature difference is the same, the output measured by the thermopile is also the same. Figure 2f, g show that the sensor output concurrently increases with the thickness of the thermal resistance layer; moreover, as the thermal conductivity of the thermal resistance layer increases, the sensor output decreases. However, the sensor thickness and thermal conductivity are generally constant values once the sensor is prepared, so they do not affect the measured heat flux. The study of the role of the thickness and thermal conductivity on the output is beneficial to sensor parameter design.

### Sensor preparation

**Fig. 3:**
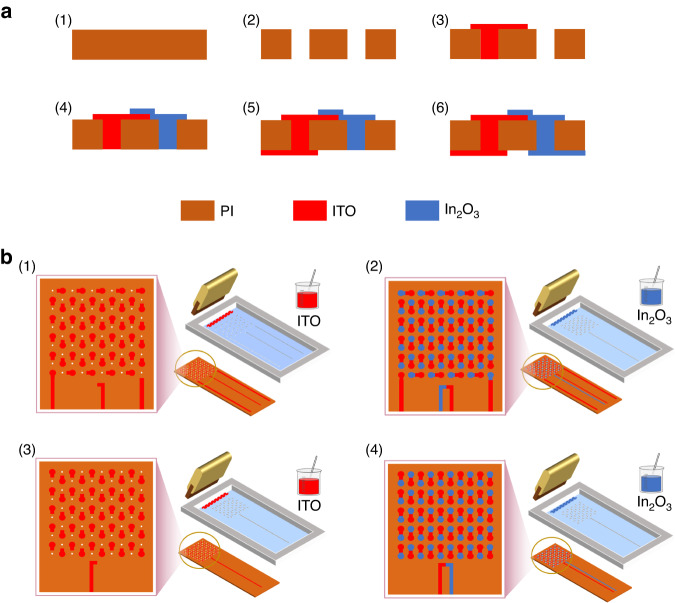
Preparation and characterization of the sensor. **a** HFS single node preparation process. **b** Preparation process of the screen printing-based HFS

The preparation process of the HFS is shown in Fig. 3a, b. Figure 3a shows the preparation process of a single node of the HFS. Figure 3a(1) shows a complete PI film, and Fig. 3a(2) shows the microhole array drilled in it. Figure 3a(3) shows printed ITO when the small hole has been filled with paste, and Fig. 3a(4) shows printed In_2_O_3_. Figure 3a(5, 6) show printed ITO and In_2_O_3_ on the other side. Figure 3b shows the preparation process of the screen printing-based HFS. To prepare the substrate, 100 0.3 mm-diameter holes were drilled in the PI film. Each small hole was spaced 2 mm apart. Then, the PI substrate was placed under the screen-printing stencil. The ITO paste was spin-coated onto the stencil and printed onto the PI substrate with a squeegee. The same method was used to print In_2_O_3_, and the substrate was flipped to the back side to print ITO and In_2_O_3_. The paste penetrated into the microhole so that the thermoelectric films on the upper and lower surfaces could be connected. Through structural design, the printed ITO-In_2_O_3_ forms a thermopile, as half of its nodes are distributed on the upper surface and the other half are on the lower surface, forming the structure required for heat flux measurement.

**Fig. 4:**
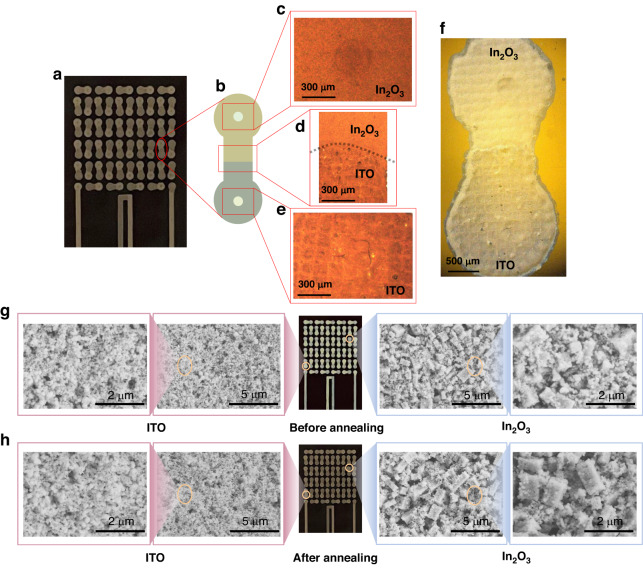
Characterization of the sensor. **a**–**f** Morphological observation under a light microscope. **g**, **h** Micromorphology characterization of the thermoelectric layer

Figure 4a–f shows images of the sensitive layer obtained under a light microscope. Figure 4a shows the sensor, and Fig. 4b shows a schematic diagram of a single node. Figure 4c, e show images of the small holes filled with In_2_O_3_ and ITO, respectively, obtained under a light microscope. The holes here do not cause the sensitive layer to come off, and the holes are filled with sensitive material to ensure the electrical connection of the whole device. Figure 4d shows an image of the lap joint of ITO and In_2_O_3_, where there is no deformation caused by the accumulation of multiple layers of material. Figure 4f shows a complete image of a single node obtained under a light microscope.

The ITO and In_2_O_3_ pastes consist of powder, epoxy resin, polyetheramine, and a solvent. The curing reaction of epoxy resin and the polyetheramine curing agent was used to generate reticulated macromolecules, thus dispersing the powder. Pine oil alcohol was used as a solvent and annealed to exclude excess organic matter so that the oxide could adhere to the flexible PI substrate. Microscopic SEM images of ITO and In_2_O_3_ before and after annealing are shown in Fig. 4g, h.

## Results

### Sensor performance test and analysis

**Fig. 5:**
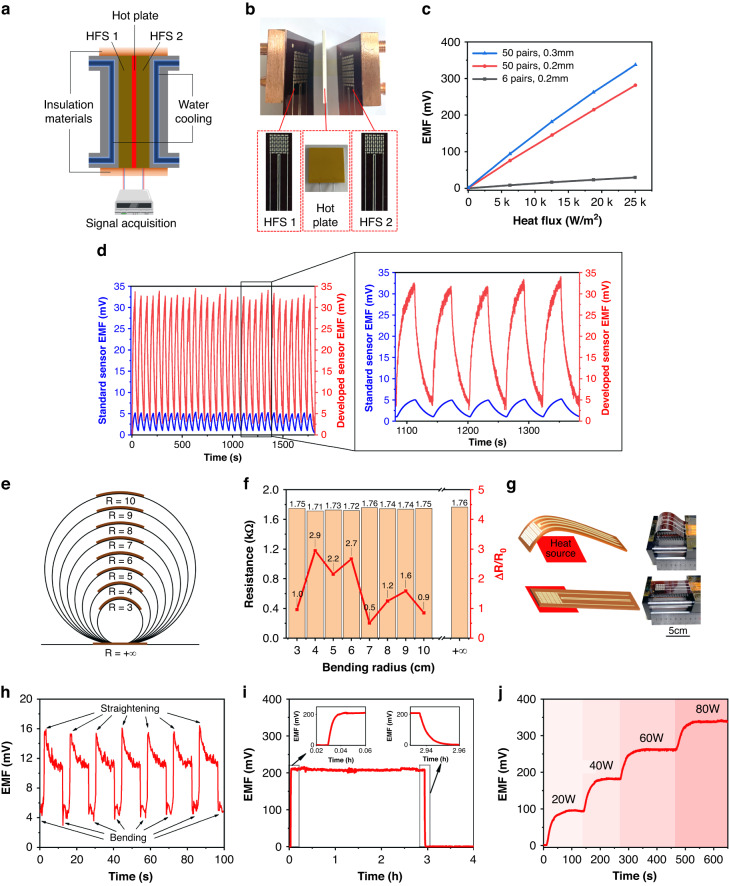
Performance testing of the HFS. **a** HFS calibration device designed based on Fourier’s law of heat conductivity. **b** Physical diagram of the calibration, including the heat source and two developed HFSs. **c** Calibration curves of HFSs. **d** HFS output voltage under 30 heat flux rise-fall cycles. **e** The HFS was bent to different radii with curvatures from 3 cm to 10 cm. **f** Resistance of the HFS with different curvatures. **g** Schematic diagram of the sensor being repeatedly stretched and bent. **h** Output voltage of the sensor under bending and stretching. **i** Long-term test of the HFS. **j** Thermoelectric output of the HFS under different heating powers. When the power is 80 W, the temperature is close to the limit of the sensor

The sensor performance is shown in Fig. 5 and Fig. S2–4. The calibration of the HFS is shown in Fig. 5a. The calibration device consists of a controllable power heating sheet, two HFSs to be calibrated, a water circulation cooling system, and a signal acquisition device. The heating sheet provides a stable heat flux source. Two identical HFSs are distributed on either side of the heating sheet. The heat of the heating sheet is uniformly transferred to both sides, and the heat flux through a single HFS can be calculated by Eq. (5).

5 $$q=\frac{P}{2A}$$

where *q* (W/m^2^) is the heat flux through a single sensor, *P* (W) is the heating power of the heating sheet, and *A* (m^2^) is the area of the heating sheet. Figure 5b shows a physical diagram of the calibration. The diagram shows the hot plate, two developed HFSs, and water-cooling system. The calibration results are shown in Fig. 5c. The blue line corresponds to the 0.3 mm thick HFS consisting of 50 thermocouples with a sensitivity coefficient of 13.43 μV/(W/m^2^), and the electromotive force-heat flux linear fitting R^2^ is 0.99485. The red line corresponds to the 0.2 mm thick HFS consisting of 50 thermocouples with a sensitivity coefficient of 11.21 μV/(W/m^2^), and the output electromotive force-heat flux linear fitting R^2^ is 0.99944. The gray line corresponds to the 0.2 mm thick HFS consisting of 6 thermocouples with a sensitivity coefficient of 1.19 μV/(W/m^2^), and the electromotive force-heat flux linear fitting R^2^ is 0.99751. The results show that the output electromotive force of the sensor is improved by increasing the number of thermocouples in series and the thickness of the HFS, which is consistent with the simulation results.

Figure 5d shows the HFS cycling experiment, in which the heat flux was repeatedly cycled from low to high and then from high to low 30 times; five of the magnified cycles are shown on the right. The red curve is the output curve of the developed HFS, and the blue curve is the output curve of a commercial heat flux sensor (HS-10B). HFS maintains a stable output in the multicycle test and has significant advantages over the output signal of the commercial sensor. The results show that ITO-In_2_O_3_ as a thermoelectric material improves the output performance of the HFS.

Figure 5e, f show the sensor bent to different curvatures. The radius of curvature was increased from 3 cm to 10 cm every 1 cm, the sensor was then flattened, and the resistance of the sensor under different curvatures was recorded. When the radius of curvature was 4 cm, the rate of change in the resistance was the largest, with a 2.9% change compared to that in the flat state. In the bent state, the change for the HFS was less than 3%; therefore, bending little affects the resistance of the HFS, showing improved flexibility characteristics of the sensor.

Figure 5g, h show the results when the sensor was bent and stretched several times. A heating sheet was placed at the bottom of the sensor as a heat source. When the sensor was bent, the sensitive part faced away from the heat source; when the sensor was straightened, the sensitive part touched the heat source. A cycle of straightening for 10 s and bending for 4 s was applied. The output signal of the HFS is shown in Fig. 5h. When the HFS is stretched flat, the output electric potential reaches a maximum of 16 mV. With heat transfer, the output gradually decreases, whereas when the HFS is bent, the output rapidly decreases. This change occurs because when bent, the sensitive part of the HFS faces away from the heat source, and heat transfer stops, so the sensor output decreases. Nevertheless, the sensor output is not reduced to zero because the heat transferred from the heat source to the sensor during stretching does not completely dissipate, and the sensor can still release heat into the air.

Figure 5i shows the thermoelectric potential output of the HFS during a long-term test. The installation of the sensor is the same as that in Fig. 5b. We recorded the output electromotive force of the sensor for approximately 3 h when the heat source was heating and for 1 h when the heat source was turned off. The maximum output voltage of the sensor is 217.0 mV, the minimum output voltage is 199.2 mV, and the average output voltage is 208.4 mV. The deviation between the maximum value and the average value is 4.13%, and the deviation between the minimum value and the average value is 4.41%, showing the stability of the sensor output. Figure 5j shows the output of the HFS under different heating powers of the heating sheet. When the power of the heating sheet is 80 W, the temperature reaches 300 °C after 100 s, which is close to the temperature resistance limit of the PI film. At this time, the heat flux reaches 25 kW/m^2^, and the HFS still maintains stability. Even if the heat flux is further increased, the sensor is expected to continue working. However, considering redundancy, the heat flux measurement limit of the HFS is 25 kW/m^2^.

**Fig. 6:**
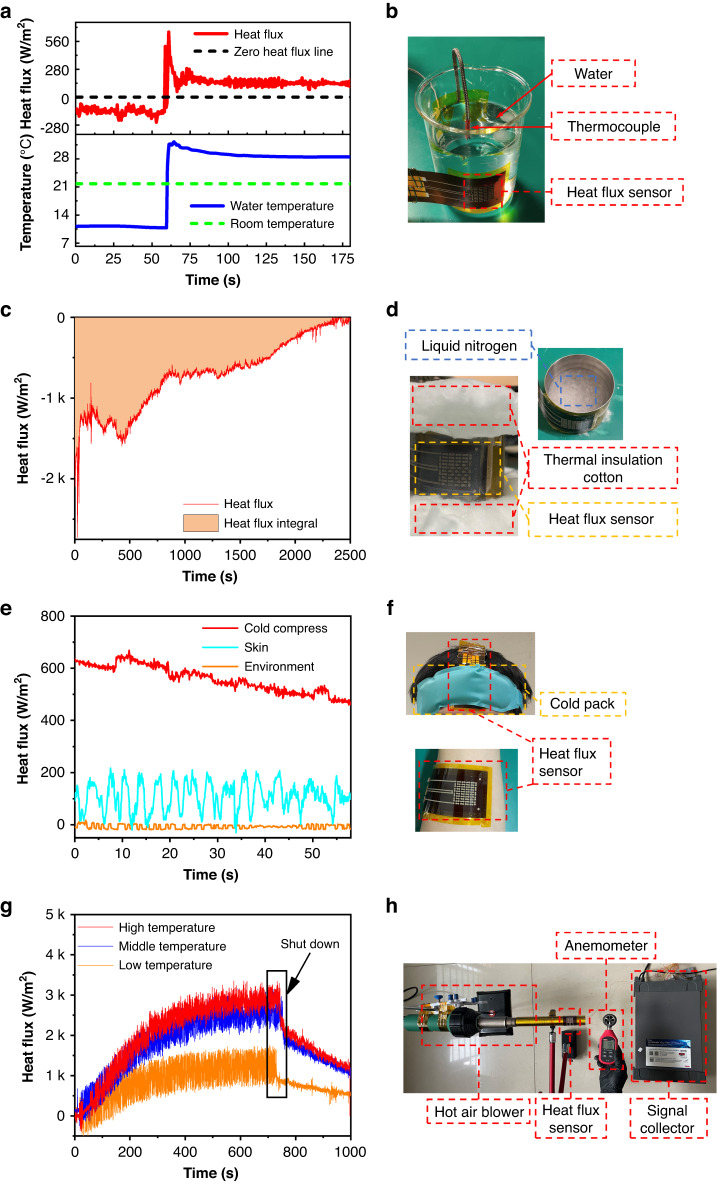
Application of the HFS for heat flux measurements. **a** Measurement of heat absorption and release of water by the HFS. **b** Pictures of heat absorption and release experiments. **c** Heat flux curve recorded by the HFS in the liquid nitrogen volatilization experiment. **d** Picture of the liquid nitrogen vaporization experiment. **e** Heat flux curve of the human body surface recorded by the HFS at room temperature and under cold application, and output curve of the HFS under no load. **f** Picture of the HFS attached to human skin. **g** Heat flux curve recorded by the HFS at different wind temperatures, recorded from when the heat gun was turned on to a period of time after it was turned off. **h** Picture of the hot air experiment

As shown in Fig. 6a, b, we utilized the HFS to measure the heat absorption and release of water. In this process, the room temperature was 21.8 °C, a standard K-type thermocouple was used to record the water temperature, and the sensor was attached to the outer wall of the beaker. First, the beaker was filled with cold water, and its temperature was maintained at 11 °C. At this time, the water absorbed external heat, and the output of the HFS was negative. After pouring hot water into the beaker, the temperature of the water rapidly rose to 32 °C and then stayed at 28.5 °C. The output of the HFS rapidly increased with increasing temperature, and the maximum value was 616 W/m^2^. When the water temperature gradually decreased and stabilized, the output of the HFS also decreased and stabilized at 125 W/m^2^. The results show that the HFS can achieve the measurement of heat absorption and heat release even for a small heat flux with a temperature difference of less than 10 °C.

As shown in Fig. 6c, d, the HFS was used to measure the endothermic process of liquid nitrogen vaporization. Fig. S3 shows the change in the sensor resistance after being placed in liquid nitrogen. As shown in Fig. 6d, the HFS was attached to the surface of the vessel containing liquid nitrogen, and heat-resistant cotton was applied to the top and bottom surfaces of the vessel to insulate and delay the vaporization process. Thus, heat is only absorbed from the side of the vessel. The heat flux of this process over time is recorded in Fig. 6c. Initially, the vessel was filled with liquid nitrogen, and violent vaporization occurred, absorbing a large amount of heat. As the liquid nitrogen volatilized, the heat absorption gradually decreased until the liquid nitrogen was completely vaporized and the heat flux became zero. The magnitude of the heat of vaporization was calculated by referring to Eq. (6).

6 $${Q}_{1}={\int }_{0}^{t}{qAdt}$$

where *Q*_1_ (J) is the heat, *q* (W/m^2^) is the heat flux measured by the HFS, *A* (m^2^) is the surface area of the container, and *t* (s) is time. The curve of the heat flux with time was recorded, integrated over time, and multiplied by the heat dissipation area to obtain the heat *Q*_1_ as 16729 J. Another method is to use the product of the heat of vaporization of liquid nitrogen and its mass to calculate its heat *Q*_2_ as 20335 J. The ratio of the two is 82.3%, mainly because the heat-resistant cotton is not completely adiabatic, causing some errors. This experiment shows that the sensor can be used at ultralow temperatures and can output a stable signal.

Figure 6e, f show the results when the HFS was used to measure the heat dissipation of the human body. The bottom line is the heat flux of the environment measured when the sensor is unloaded, and the light blue line is the heat flux measured by the sensor when it is applied to the surface of the human body. Due to the influence of human sweat, the measured data have large fluctuations, with an average value of 108 W/m^2^. The red line is the heat flux measured when a cold pack was applied to the forehead. During the cold compress process, the human body heat flux reached a maximum of 669 W/m^2^, and its gradually decreased as the compress interface gradually reached thermal equilibrium.

Figure 6g, h show the results when the HFS was used to measure the heat dissipation in a hot air tube. Hot air was generated by a hot air blower and then blown into the round tube, the surface of which had an HFS attached. The wind speed was measured using an anemometer, and the signal acquisition device collected the sensor output signal. The heat gun was set to low temperature, medium temperature, and high temperature, and the sensor output was recorded. The orange line is the output under low-temperature wind (141 °C). When the heat transfer was stable, the output was stable at approximately 1272 W/m^2^. The blue line shows the output under medium-temperature wind (252 °C). When the heat transfer was stable, the output was stable at approximately 2512 W/m^2^. The red line shows the output under high-temperature wind (276 °C). When the heat transfer was stable, the output was stable at approximately 2808 W/m^2^.

## Discussion

The experiments verified the adaptability of the sensor to high and low temperatures. The HFS showed high sensitivity and thermostability. The heat transfer process can be analyzed based on the output values of the sensor. These values can be used in the determination of the thermal power of electronic components and to provide data support for the study of devices with low heat generation and power consumption. This method can be applied to wearable devices to measure human heat dissipation and is useful for studying human diseases related to heat generation. However, there are still several problems that need to be solved; for example, the accuracy of the sensor in low-temperature measurements needs to be further improved, but the sensor is still relevant for heat transfer process measurements.

## Conclusion

In this study, we developed a thermoelectric sensor for heat flux measurement over a large temperature range, which has a series connection thermopile created by punching a microhole array. ITO and In_2_O_3_ with high Seebeck coefficients were selected as thermoelectric materials, and the HFS was prepared with the screen-printing method. Through simulation analysis, we reveal that the thermoelectric potential output is proportional to the heat flux and independent of the temperature magnitude. The thickness and substrate thermal conductivity of the thermoelectric sensor affect the parameters of the sensor. The sensor exhibits excellent flexibility, with a resistance change of less than 3% during bending to a radius of 3 cm. Through calibration, we found that the sensitivities of HFSs with thicknesses of 0.2 mm and 0.3 mm reach 11.21 μV/(W/m^2^) and 13.43 μV/(W/m^2^), respectively. The HFS can measure heat flux up to 25 kW/m^2^ and successfully measure the heat flux outputs from cold or hot water, liquid nitrogen volatilization (−196 °C), human skin heat dissipation, and hot air (276 °C). In other words, the sensor can withstand both ultralow and high temperatures, has high flexibility and sensitivity, and can measure both heat absorption and exothermic effects. This sensor has great potential for applications in the superconductor and cryobiology fields.

### Supplementary information

Supplemental Material
