# Supplementary material for: Highly sensitive flexible heat flux sensor based on a microhole array for ultralow to high temperatures
Source: Microsyst Nanoeng. 2023 Oct 24;9:133. doi: 10.1038/s41378-023-00599-9 (PMC10598026; doi:10.1038/s41378-023-00599-9)
Supplement: Supplementary file 1 — Supplemental Material [file 41378_2023_599_MOESM1_ESM.docx]

**Supplementary Information**

**Highly sensitive flexible heat flux sensor based on a microhole array for ultralow to high temperatures**

Le Li^1^, Bian Tian^1,2,3,*^, Zhongkai Zhang^1,*^, Meng Shi^1^, Jiangjiang Liu^1^, Zhaojun Liu^1^, Jiaming Lei^1^, Shuimin Li^1^, Qijing Lin^1^, Libo Zhao^1^ and Zhuangde Jiang^1^

*^1^* *School of Mechanical Engineering, Xi’an Jiaotong University, 710049 Xi’an, China*

*^2^* *State Key Laboratory for Manufacturing Systems Engineering, International Joint Laboratory for Micro/Nano Manufacturing and Measurement Technologies, Xi’an Jiaotong University (Yantai) Research Institute for Intelligent Sensing Technology and System, Xi'an Jiaotong University, Xi'an 710049, China*

*^3^* *Shandong Laboratory of Yantai Advanced Materials and Green Manufacturing, Yantai 265503, China*

*Correspondence*:* *Bian Tian (*[*t.b12@mail.xjtu.edu.cn*](mailto:t.b12@mail.xjtu.edu.cn)*) or Zhongkai Zhang (zhangzk@xjtu.edu.cn)*

Fig. S1 shows the calculation process by Comsol Multiphysic. Fig. S1a shows the established model, and Table S1 shows the main geometric parameters. Fig. S1b shows the domain contained in the physical field of the thermoelectric effect. Fig. S1c shows the boundary of the applied temperature, which is the model's upper surface. The boundary of the heat flux includes the upper and lower surfaces. Fig. S1d, S1e, and S1f show the material settings of each part. Fig. S1d shows ITO, which is distributed on the upper surface, on the lower surface, inside the small hole, and on the thermocouple part. Fig. S1e shows In_2_O_3_, and Fig. S1f shows PI.


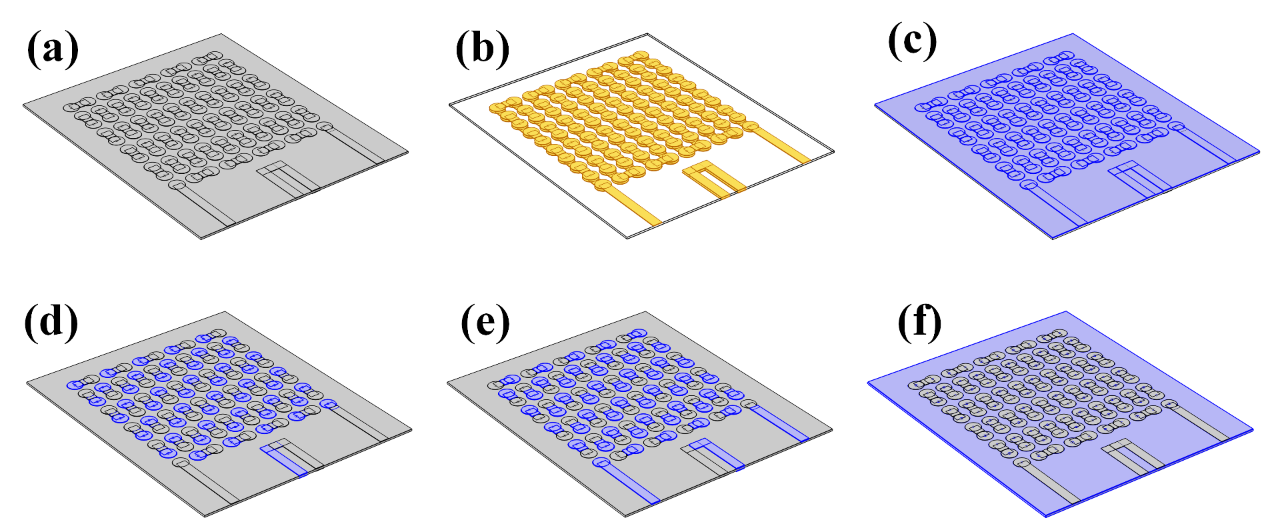


**Fig. S1 Sensor simulation calculation process.** **a** The established model. **b** Physical field of thermoelectric effect. **c** Temperature boundary. **d** Domain contained by ITO material. **e** Domain contained by In_2_O_3_ material. **f** Domain contained by PI material.

**Table S1 Main geometric parameters of the sensor**

| **Component** | **Characteristics** | **Values (mm)** |
| --- | --- | --- |
| Substrate/thermal resistance layer | length | 30 |
|  | width | 25 |
|  | thickness | 0.2 |
| thermoelectric layer | thickness | 0.03 |
|  | width | 1 |
|  | Diameter at disk | 1.6 |
|  | Diameter of the small hole | 0.3 |

Fig. S2 shows the flexibility of the sensor on a shaped surface. Fig. S2a shows the shape of the sensor in the bent state. According to the experiments in this paper, the resistance of the sensor changes by less than 3%. Fig. S2b and S2c show the HFS attached along the direction perpendicular and parallel to the bus of the cone. Fig. S2d shows the HFS apposition on the cylindrical surface. Fig. S2e shows the shape and quality of the HFS. The heat flux sensitive area is 2 cm × 2 cm. When the thickness of the sensor base is 0.2 mm, the total thickness is 0.26 mm, and the mass is 1.368 g.


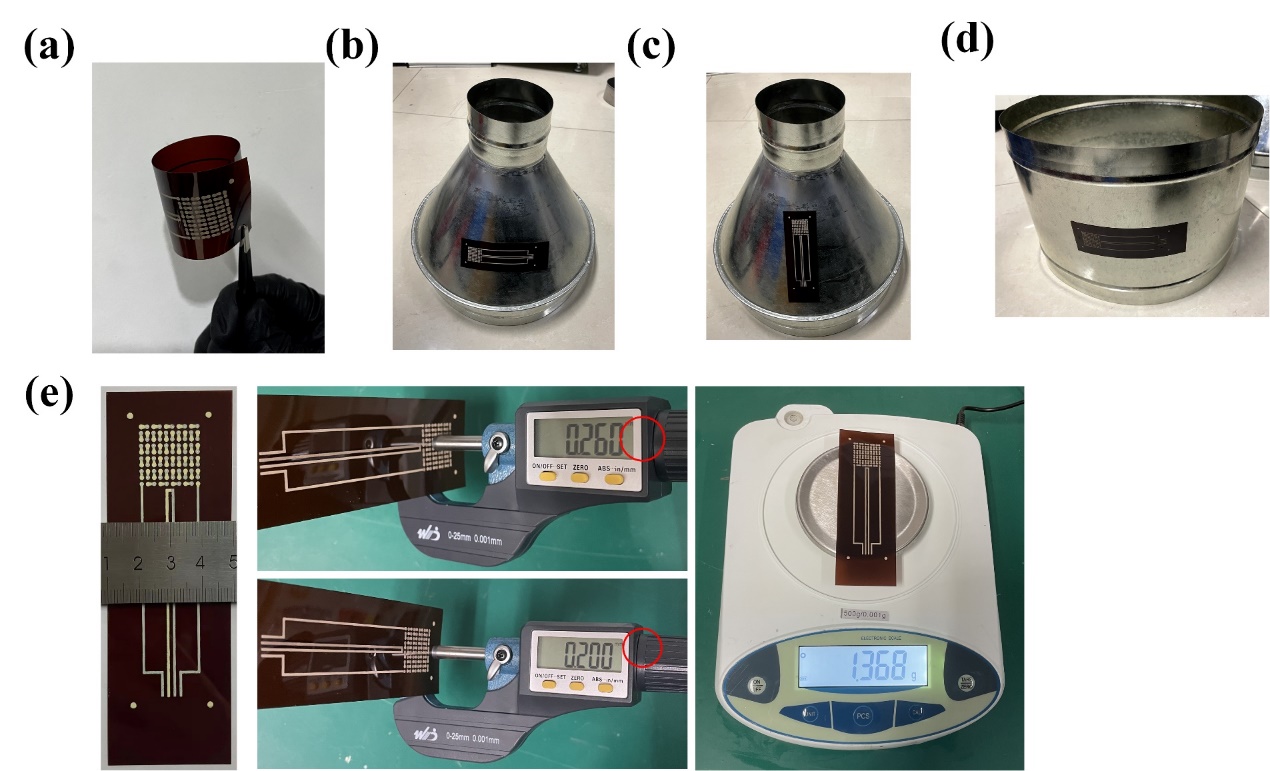


**Fig. S2** Sensor attachment on curved surfaces and the characteristics.

Fig. S3 shows the sensor in the liquid nitrogen infiltration process. A sufficient amount of liquid nitrogen is poured into the iron box. The sensor is placed in liquid nitrogen for 10 seconds, the sensor did not break or shrink after taking out.

The resistance value of the HFS is 1.65 MΩ before putting in and 1.71 MΩ after putting in, and the resistance values of the two thermocouples are 0.56 MΩ and 0.61 MΩ before putting in and 0.56 MΩ and 0.60 MΩ after putting in.


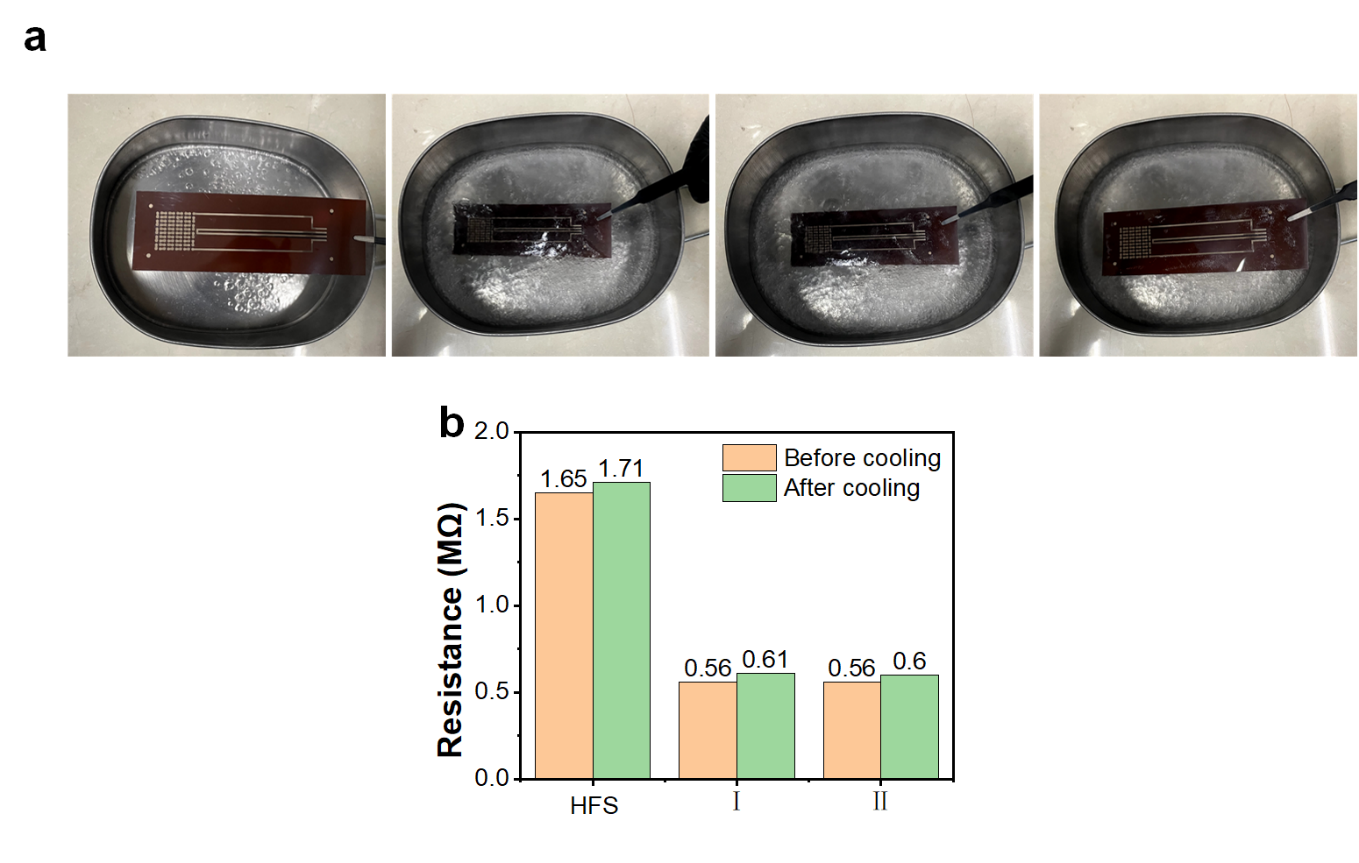


**Fig. S3** Sensor resistance change before and after being placed in liquid nitrogen.

Fig. S4 shows the performance of ITO-In_2_O_3_ as a thermoelectric material. The calibration process is as shown in Fig. S4a, the temperature-sensitive part of the sensor is heated with a heating pad, and the temperature of the heating pad is controlled to make its temperature rise slowly. The temperature of the hot end is recorded using a standard thermocouple. The Seebeck coefficient is calculated as 127.8 μV/℃, and the R^2^ of linear fit to temperature is 0.99864.


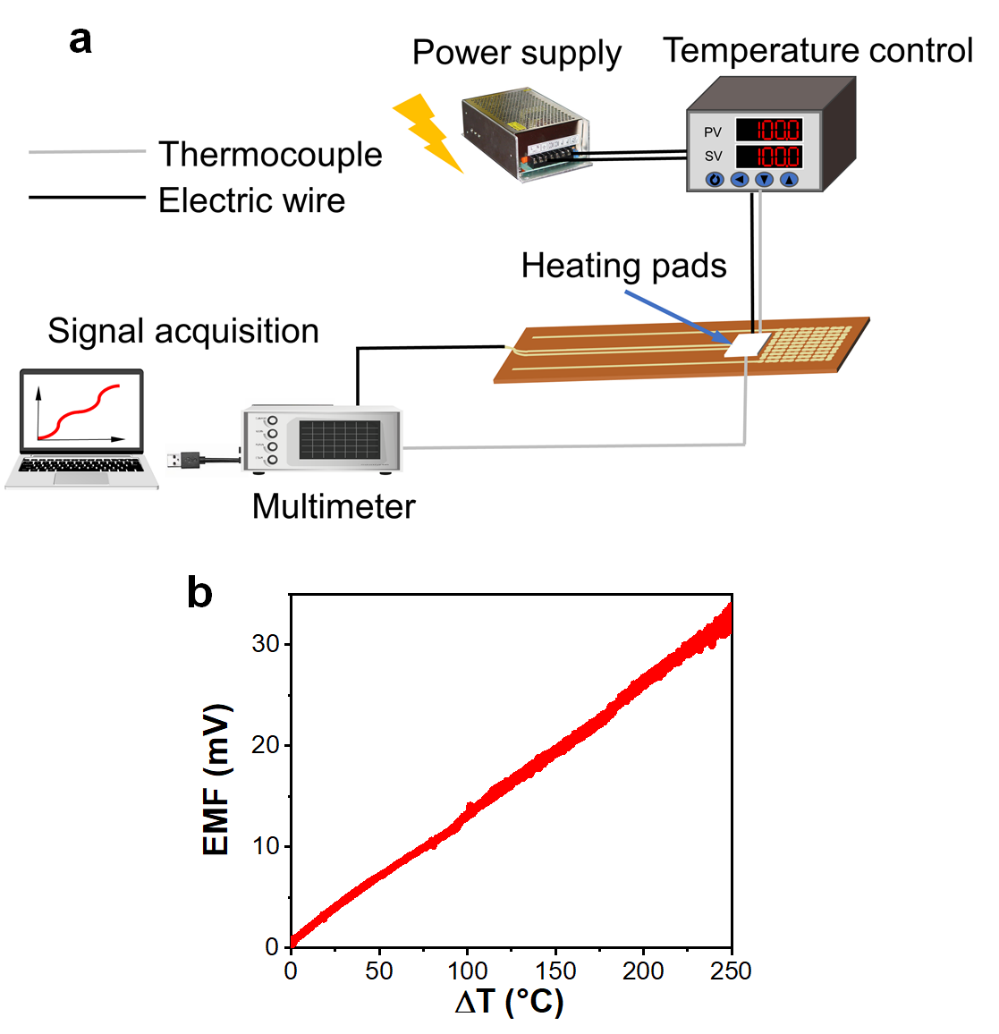


**Fig. S4** Calibration and results of ITO-In_2_O_3_ temperature sensor.

**References**

1. Zhao, T. Y. *et al.* Experiments and COMSOL simulations: A comparative study of the heat flux plate method and the gradient method for soil heat flux measurements in barren sand. *Agr Forest Meteorol* **334** (2023).
2. Zhao, X. H., Li, H. T., Jiang, S. W., Zhang, W. L. & Jiang, H. C. Effect of nitrogen doping on the thermoelectric properties of ITO-In2O3 thin film thermocouples. *Thin Solid Films* **629**, 1-5 (2017).
